# Subdural anaplastic large-cell lymphoma presenting as a subacute epidural hematoma on imaging: A case report

**DOI:** 10.1097/MD.0000000000030012

**Published:** 2022-08-05

**Authors:** Tingting Yu, Jibo Yin, Hongyue Huo, Haixue Zhao, Zhongwen Wang, Jianxin Jiang

**Affiliations:** a Graduate School of Dalian Medical University, Lvshunkou Kou District, Dalian, Liaoning, China; b Department of Neurosurgery, Taizhou People’s Hospital affiliated to Nanjing Medical University, Hailing District, Taizhou, Jiangsu, China; c Department of Neurology, Taizhou People’s Hospital, Hailing District, Taizhou, Jiangsu, China; d Department of Neurosurgery, Lufeng People’s Hospital, Chuxiong State, Yunnan Province, China; e Department of Neurosurgery, Taizhou People’s Hospital, Hailing District, Taizhou, Jiangsu, China.

**Keywords:** case report, central nervous system, imageology, subdural anaplastic large-cell lymphoma, subdural lymphoma

## Abstract

**Rationale:**

Subdural anaplastic large-cell lymphoma (SALCL) is an extremely rare subtype of primary central nervous system (CNS) lymphoma. Here, we report a very rare subdural lymphoma case, which was misdiagnosed as a subacute epidural hematoma based on the radiological examination.

**Patient concerns:**

We present the case of an 82-year-old patient who presented with a 2-day history of headache and consciousness disorder following head injury. Computed tomography of the head revealed a fusiform isodense/slightly dense shadow under the right temporoparietal occipital cranial plate, suggesting a subacute epidural hematoma. It was initially misdiagnosed as a right traumatic subacute epidural hematoma with hemiplegia of the left limb. According to the patient’s condition, an emergency craniotomy was performed to remove the hematoma. However, it was found that the lesion was located under the dura mater and was yellowish-brown with yellowish-brown liquid inside. The appearance of the lesion looked like bean curd residue. Histopathological examination diagnosed ALCL.

**Diagnosis:**

SALCL presenting as a subacute epidural hematoma on imaging.

**Interventions:**

Operation.

**Outcomes:**

The patient died 1 month after being discharged automatically.

**Conclusions:**

This report shows a rare radiography presentation of SALCL. SALCL can mimic the appearance of an epidural hematoma and should be regarded as a differential diagnosis even in patients with a history of craniocerebral injury and the “typical” imaging appearance of an epidural hematoma. The report is hoped to provide a scientific reference for the clinical diagnosis of subdural lymphoma.

## 1. Introduction

Central nervous system (CNS) lymphomas include primary CNS lymphomas and systemic lymphomas that invade the CNS as secondary lymphomas, accounting for 1% to 3% of CNS tumors. Subdural lymphoma is rarer, and the cause of the disease is unknown. Lymphoma of the CNS has been reported to be common in the cerebral parenchyma and partly involves the meninges. In primary CNS lymphomas, diffuse large B-cell lymphomas account for >90% of primary CNS lymphomas, and T-cell lymphomas account for <5%.^[1,2]^ Anaplastic large-cell lymphoma (ALCL) is a rare type of non-Hodgkin’s lymphoma (NHL) and a subtype of T-cell lymphoma, accounting for about 1% to 3% of NHL and 15% of T-cell lymphoma. ALCL often occurs in lymph nodes and extranodal lymphoid tissues, commonly in the skin, bone, soft tissues, and other parts outside the node, and rarely in the brain, bronchi, and other parts.

ALCL is rarely reported, and the imaging manifestations are nonspecific, clinically easy to misdiagnose, and the exact diagnosis of this disease depends on pathological histology. Until now, the understanding of this lymphoma is not profound enough. Herein, a clinically rare case of subdural ALCL whose imaging appearance resembles an epidural hematoma is reported. The report is as follows.

## 2. Case report

**Figure 1.**
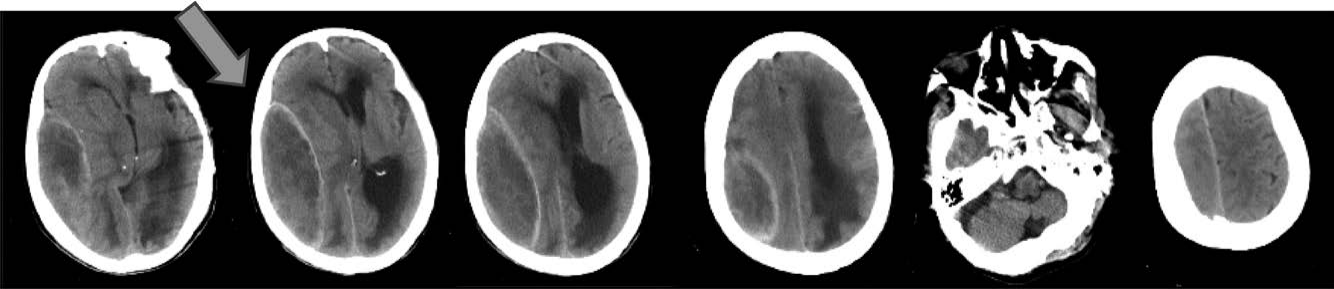
Preoperative noncontrast CT scan of the patient’s head. Fusiform isodense/slightly dense shadow was seen under the right temporoparietal occipital cranial plate, which was considered a subacute epidural hematoma. The maximum layer was 117 mm×42 mm, and the thickness of the 10 mm layer covered 7 layers. The right lateral ventricle was compressed. The midline of the brain shifted to the left. CT = computed tomography.

An 82-year-old man presented to the hospital with “headache and consciousness disorder for 2 days after head injury.” He has a history of hypertension. The patient’s family complained that the patient fell and hurt his head 2 days ago, and then the patient appeared to have unclear speech, unable to answer questions correctly, accompanied by blurred consciousness, accompanied by weakness of the left limb activity, unconscious disorder, limb convulsion, no nausea, vomiting, no incontinence, no systematic diagnosis and treatment, and the degree of consciousness disorder increased and came to the hospital. Physical examination on admission included temperature: 36.7°C, pulse: 66 times/min, respiratory rate: 19 times/min, blood pressure: 76/92 mm Hg, Glasgow Coma Score (E2-V4-M4) 10 points, blurred consciousness, incomplete mixed aphasias, no malformation of skull appearance, no swelling, tenderness, and no skull depression touched. Bilateral pupils were equal in size and circle, with a diameter of 2 mm. Bilateral pupils are insensitive to light. The muscle strength of the left limb was level 0, with slightly higher muscle tension, and the right limb had involuntary activity. The muscle strength was level 3, the muscle tension was normal, and the bilateral Babinski sign was positive. The breath sounds of both lungs were coarse, and a few moist rales could be heard at the bottom of the lungs. Emergency head computed tomography (CT) scan showed fusiform density under the right temporoparietal occipital cranial plate, obvious midline deviation, and ventricle compression. The subacute epidural hematoma was considered (Fig. 1).

**Figure 2.**
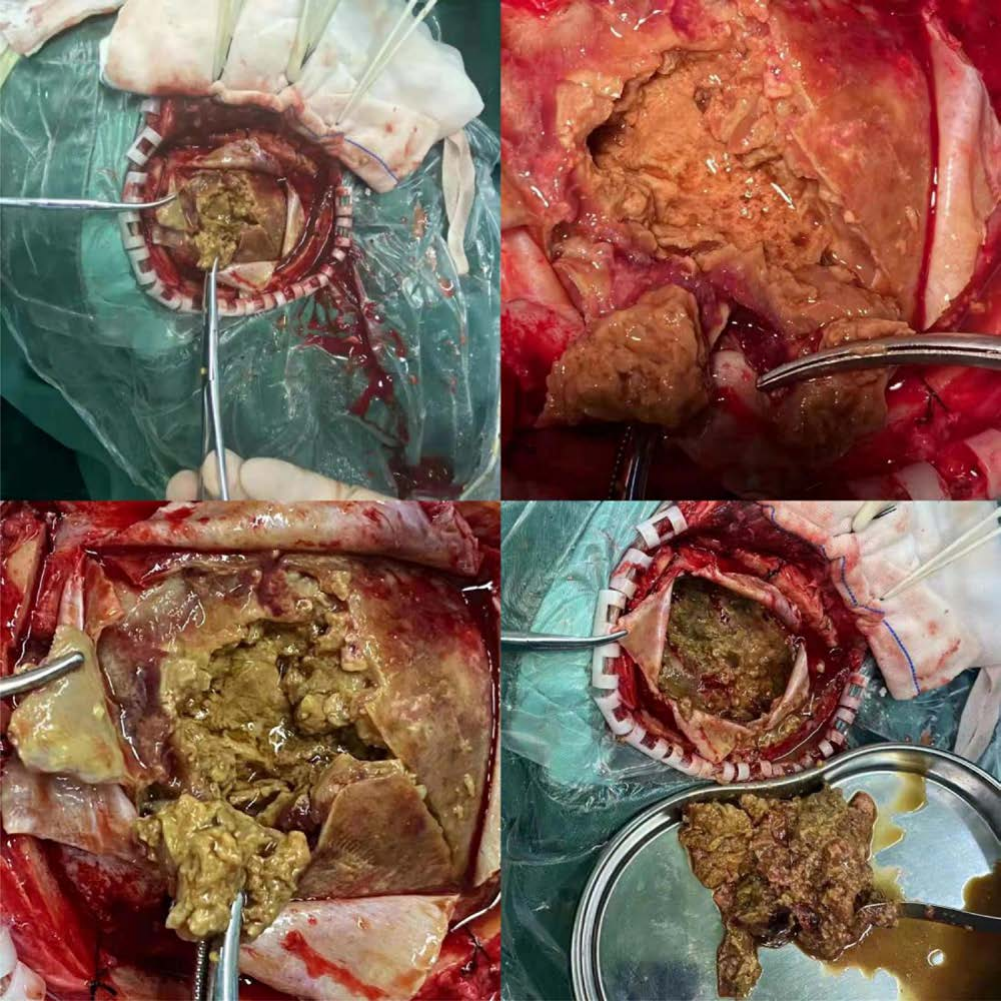
Intraoperative picture. The yellow-brown lesion was like tofu residue, and yellow-brown fluid was seen in the middle. The subdural tofu residue was cleared and occupied about 170 g.

**Figure 3.**
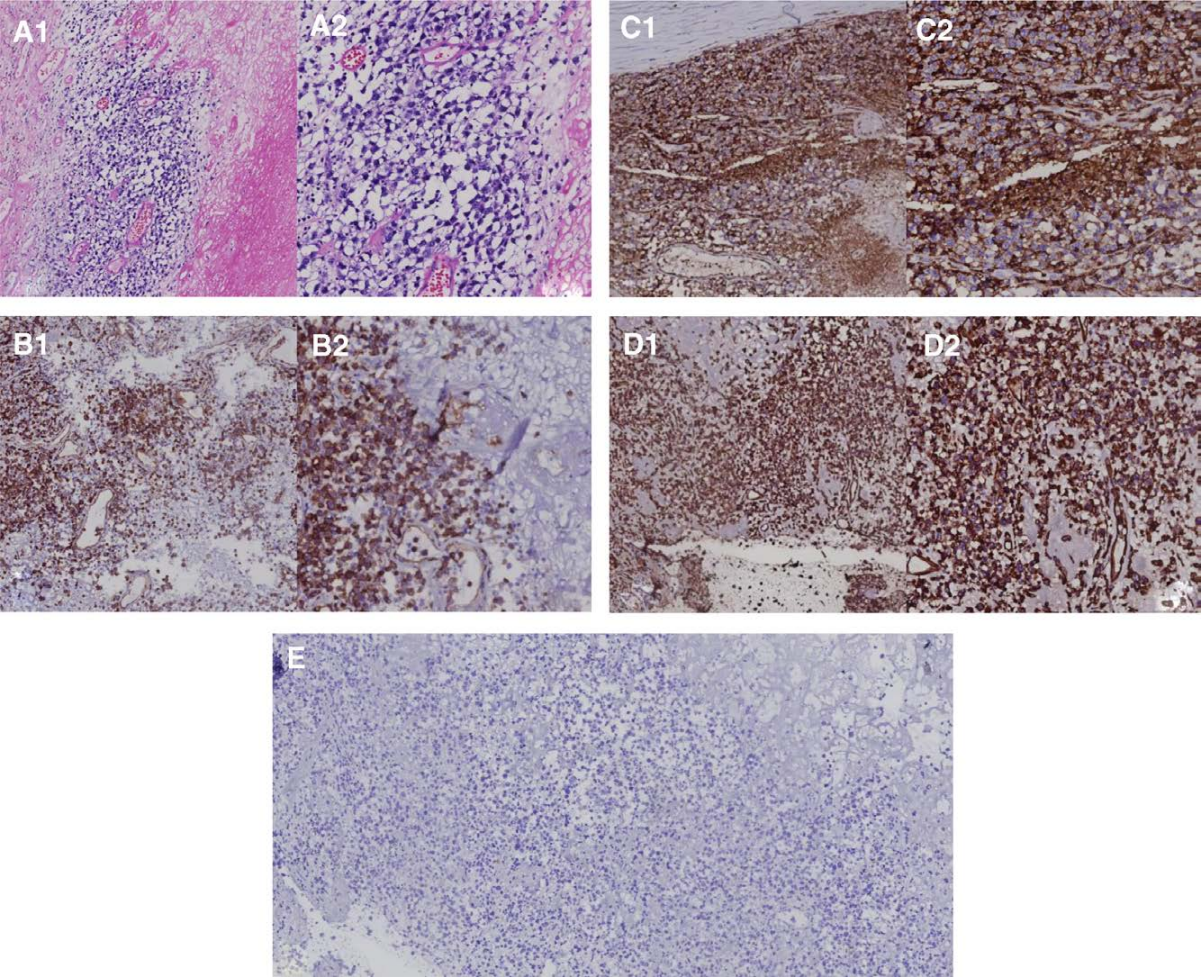
Postoperative pathological. Right temporal–parietal malignant tumor with extensive necrosis was considered anaplastic large-cell lymphoma combined with immunohistochemical results. Immunohistochemical results: LCA, CD3, ALK, CD30, TIA-1, and Vimentin are positive; enzyme B is scattered positive; ki-67 (90%) is positive. Note: The tumor cells, in this case, were mainly positive for T cells, ALK, and CD30. It was relatively supportive of anaplastic large-cell lymphoma combined with the morphology. However, abnormal expression of cytotoxic markers is rare. (A1) hematoxylin and eosin, original magnification ×10. (A2) hematoxylin and eosin, original magnification ×20. (B1) CD3(original magnification ×10. (B2) CD3 (original magnification ×20). (C1) LCA (original magnification ×10) (C2) LCA (original magnification ×20). (D1) VIM (original magnification ×10). (D2) VIM (original magnification ×20). (E) CK (original magnification ×10).

After admission, emergency removal of right temporoparietal occipital hematoma was performed under general anesthesia. During the operation, yellow-brown lesions were observed in the subdural area, which looked like tofu residue, and yellow-brown fluid has seen on the lesion. About 200 g of subdural tofu residue was removed (Fig. 2). Postoperative pathological sections were sent to Nanjing First Hospital for consultation, and the results showed that the right temporal–parietal malignant tumor was accompanied by extensive necrosis. Combined with the immunohistochemical results (tumor cells mainly expressed T cells; ALK and CD30 were positive), ALCL was considered. However, abnormal expression of cytotoxic markers was rare (Fig. 3). After surgery, the patient was transferred to the department of the intensive care unit for treatment. The patient’s consciousness improved with a Glasgow Coma Score of 12, and he could be woken up but could not answer. The muscle strength of his limbs did not improve significantly. During the treatment, a pulmonary infection happened in the patient. On the seventh day after the operation, the patient had blurred consciousness, poor food intake, and left limb muscle strength of grades 3 to 4. Due to a severe pulmonary infection, his family refused further treatment and discharged him. Unfortunately, the patient eventually died about a month after discharge.

## 3. Discussion

In 1985, Stein et al^[3]^ proposed that ALCL is cytological that strongly expresses Ki-1. ALCL accounts for about 10% to 15% of NHL in children and 3% in adults.^[4]^ It involves lymph nodes and external nodes such as skin, soft tissue, lungs, bone, and liver. Located in the CNS, whether primary or secondary, it is extremely rare. Relatively speaking, secondary CNS involvement is not uncommon, with a reported incidence of 5% to 11%.^[5]^ In these cases, B-cell lymphoma predominates, with priority in the skull, more often in the dura and meningeal cartilage and the parenchyma, while a large amount of spinal involvement is intramedullary or epidural.^[6,7]^ The lack of clinical and radiographic features that typically distinguish common subdural/epidural lesions (such as bleeding, infection, and hydro max) from less common lesions (tumors) poses significant challenges for clinical management. Fusiform masses at the cranial top can be associated with a range of neurological deficits and can be easily identified by routine plain CT. In theory, any subdural/extradural mass can attenuate the X-ray and reproduce the image associated with the hematoma. A mass with a high attenuation coefficient can simulate an acute blood clot, while a mass with a low attenuation coefficient can simulate a liquefied clot. It has been reported that the imaging characteristics of dural lymphoma are similar to those of subdural hematoma. However, in this case, subdural lymphoma mimics subacute epidural hematoma on head CT imaging features. In this case, the following aspects are special. The first is the location of the lymphoma. Head noncontrast CT scan imaging features of subdural lymphoma are similar to the subacute epidural hematoma in this case. A search of the literature revealed that dural lymphoma often mimics the clinical presentation of a subdural hematoma.^[8]^ The second is the type of lymphoma. At present, there are mainly the following types reported: lymphoplasmacytic lymphoma,^[9]^ marginal zone lymphoma,^[10–13]^ diffuse B-cell lymphoma,^[14]^ and Burkitt lymphoma.^[15]^ However, large-cell lymphoma (T-cell and ALK are positive) is rare.

Since the dura does not contain any lymphoid tissue, the pathogenesis of primary subdural lymphoma remains unclear. Surgery, radiation, and chemotherapy can be recommended, but there are no standard treatment guidelines. At present, the treatment of subdural lymphoma mainly includes surgical resection combined with chemotherapy or radiotherapy, which has been reported to achieve significant results.^[10,12,13]^ However, due to the lack of reliable data for ALCL/T-cell lymphoma and the difficulty of clinical trials for CNS ALCL/T-cell lymphoma, there is still a lack of standardized treatment for CNS ALCL/T-cell lymphoma, the treatment strategy commonly used for B-cell lymphoma. At present, high-dose methotrexate (>3 g/m^2^) and rituximab should be part of any induction therapy, which can improve the response rate and overall survival rate of primary CNS diffuse large B-cell lymphoma patients. Current protocols for induction include procarbazine, temozolomide, rituximab and high-dose methotrexate/cytarabine, or prednisone, depending on geographic area and physician preference. The recurrence rate is close to 50%, and 10% to 15% of patients had primary drug resistance.^[16]^ Patients with recurrent refractory primary CNS diffuse large B-cell lymphoma have a very poor prognosis. It has become a research hotspot in recent years to apply new targeted drugs to improve the prognosis of patients with recurrent refractory type CNS lymphoma by studying gene mutations and signal transduction pathways to find new therapeutic targets, such as Bruton tyrosine kinase, mammalian target of rapamycin, immune checkpoint, and phosphatidylinositol 3-kinase inhibitors, and immunomodulators, have been used in the treatment of recurrent, refractory primary CNS diffuse large B-cell lymphoma.^[17]^

In summary, when the patient’s complete nervous system examination, head trauma history, and head CT imaging characteristics are inconsistent, if conditions permit, further imaging examination can be carried out to help diagnose. Emergency surgery is a choice when the patient’s condition does not allow it. If the imaging manifestation is considered to be epidural/subdural hematoma, but the clinical data and further imaging examination do not support it, if the space-occupying effect of the lesion is not obvious and the clinical symptoms are mild, it can be observed based on dynamic imaging examination. Make the best treatment plan according to the observation results. If the patient has the indication of emergency operation at admission, surgical treatment should be taken based on other imaging examinations at night. If necessary, an intraoperative pathological examination can be performed.

## 4. Conclusions

In this report, we present an extremely rare and atypical case of a patient with subdural ALCL presenting as a subacute epidural hematoma on imaging. The reason for such misdiagnosis is that primary lymphoma of the CNS is rare and prone to misdiagnosis. To some extent, the clinical examination is not comprehensive and careful, and the clinical analysis is not deep enough and thorough. In the future clinical diagnosis of primary CNS lymphoma, it is necessary to further clinical examination and diagnosis to avoid misdiagnosis.

## Author contributions

All authors contributed to the execution of this work and the preparation of this manuscript. All authors have read and agreed to the published version of the manuscript.
